# Measurement of Serum Melatonin in Intensive Care Unit Patients: Changes in Traumatic Brain Injury, Trauma, and Medical Conditions

**DOI:** 10.3389/fneur.2014.00237

**Published:** 2014-11-17

**Authors:** Marc A. Seifman, Keith Gomes, Phuong N. Nguyen, Michael Bailey, Jeffrey V. Rosenfeld, David J. Cooper, Maria Cristina Morganti-Kossmann

**Affiliations:** ^1^National Trauma Research Institute, The Alfred, Melbourne, VIC, Australia; ^2^Department of Surgery, Monash University, Melbourne, VIC, Australia; ^3^Department of Neurosurgery, The Alfred, Melbourne, VIC, Australia; ^4^Department of Epidemiology, Monash University, Melbourne, VIC, Australia; ^5^Australian New Zealand Intensive Care Research Centre, Melbourne, VIC, Australia; ^6^Intensive Care Unit, The Alfred, Melbourne, VIC, Australia; ^7^Department of Child Health, Barrow Neurological Institute, University of Arizona, Phoenix, AZ, USA

**Keywords:** melatonin, TBI, trauma, intensive care unit, circadian

## Abstract

Melatonin is an endogenous hormone mainly produced by the pineal gland whose dysfunction leads to abnormal sleeping patterns. Changes in melatonin have been reported in acute traumatic brain injury (TBI); however, the impact of environmental conditions typical of the intensive care unit (ICU) has not been assessed. The aim of this study was to compare daily melatonin production in three patient populations treated at the ICU to differentiate the role of TBI versus ICU conditions. Forty-five patients were recruited and divided into severe TBI, trauma without TBI, medical conditions without trauma, and compared to healthy volunteers. Serum melatonin levels were measured at four daily intervals at 0400 h, 1000 h, 1600 h, and 2200 h for 7 days post-ICU admission by commercial enzyme linked immunosorbent assay. The geometric mean concentrations (95% confidence intervals) of melatonin in these groups showed no difference being 8.3 (6.3–11.0), 9.3 (7.0–12.3), and 8.9 (6.6–11.9) pg/mL, respectively, in TBI, trauma, and intensive care cohorts. All of these patient groups demonstrated decreased melatonin concentrations when compared to control patients. This study suggests that TBI as well as ICU conditions, may have a role in the dysfunction of melatonin. Monitoring and possibly substituting melatonin acutely in these settings may assist in ameliorating long-term sleep dysfunction in all of these groups, and possibly contribute to reducing secondary brain injury in severe TBI.

## Introduction

Traumatic brain injury (TBI) remains a significant medical concern worldwide as a considerable cause of permanent disability, imposing a significant burden on society. Despite improvements in neurosurgery and intensive care treatment, mortality in severe TBI patients remains high (1, 2).

A common consequence of brain trauma is sleep disturbance including insomnia and altered sleep–wake cycles (3, 4). In the long term, sleep disturbance is associated with fatigue, depression, anxiety, and cognitive dysfunction likely due to lower evening melatonin levels (5, 6). Deficiencies in sleep may also impair recovery from brain injury by increasing catabolic rate, decreasing cellular and humoral immunity, and impairing cell division and thus compromising quality of life (7). Sleep duration and quality in intensive care unit (ICU) patients has been linked to abnormal melatonin production (7, 8). Endogenous melatonin is produced by the pineal gland and regulates the sleep–wake cycle (9, 10). The blood concentrations of melatonin increase between 0200 h and 0400 h (11) and decrease to a nadir during daylight hours. When administered, exogenous melatonin is able to induce phase shifts of the circadian rhythm of the sleep–wake cycle (10, 12).

In addition to its physiological roles in regulating sleep patterns, melatonin has been demonstrated to provide neuroprotective effects in brain injury models due to its pleiotropic properties (13–15). Its therapeutic potential has been shown to provide protection by stabilizing endothelial permeability and limiting the extent of brain edema and by reducing oxidative stress and neuronal death following injury (16–19).

The above findings have prompted the hypothesis that therapeutic application of melatonin might improve patient recovery by restoring the circadian rhythm of the sleep–wake cycle and counteract the neurotoxic biochemical cascades leading to secondary brain damage. We previously published data showing that increased cerebrospinal fluid levels of melatonin were associated with heightened isoprostane levels, a biomarker of oxidative stress, suggesting that a possible role of melatonin to combat oxidative stress (20). However, serum concentrations of melatonin measured only at one time point daily were found unchanged. Thus, to deepen our understanding on the impact of TBI on melatonin production, we embarked on a study to measure melatonin levels over four time intervals during the day and elucidate whether their alterations are specifically attributable to brain injury and/or to critical care circumstances.

## Materials and Methods

### Patients and blood sampling

This study was conducted in accordance with the National Health and Medical Research Council of Australia national statement on ethical conduct in research involving human beings and has been approved by The Alfred Hospital Human Ethics Committee. Informed consent was waived on admission and delayed written consent was obtained from either the patient or the next of kin.

Forty-five patients admitted to the ICU at The Alfred, Melbourne, were recruited to one of three groups: patients with severe TBI defined as a Glasgow coma scale (GCS) score of <9 on admission, patients having sustained extra-cranial traumatic injury but no brain injury, and patients admitted to the ICU for conditions other than trauma. Healthy volunteers were used for control measures. Details of the patients in terms of demographics and GCS are given in Table 1.

**Table 1:** **Epidemiological characteristics including age, gender, GCS, and GOSE where relevant, and mean/range of melatonin levels in three patient groups: severe TBI; trauma without severe TBI; medical conditions without trauma or TBI (demographics)**.

	TBI	Trauma without TBI	ICU	Overall
Number of patients	18	14	13	45
Age
Mean ± SEM	35.1 ± 3.2	50.7 ± 4.9	43.9 ± 4.9	42.5 ± 2.6
(range)	(16–73)	(21–86)	(22–74)	(16–86)
Gender
Male (%)	14 (78%)	13 (93%)	7 (54%)	34 (76%)
Female (%)	4 (22%)	1 (7%)	6 (46%)	11 (24%)
GCS	5.5 ± 0.8	13.2 ± 0.7		
GOSE	4.2 ± 0.4	5.4 ± 0.5		
Melatonin (pg/mL)
Geometric mean	8.3	9.3	8.9	8.9
(95% CI)	(6.3–11.0)	(7.0–12.3)	(6.6–11.9)	(7.4–10.7)

*Melatonin production over four intervals per day for seven consecutive days post-admission in ICU was calculated and showed no statistical difference among the groups (*p* = 0.87)*.

Upon recruitment, all patients underwent serial serum at 0400 h, 1000 h, 1600 h, and 2200 h. Sampling began at the next scheduled interval once the patient had been recruited. The serum samples were centrifuged for 10 min at 4°C at 200 × *g* and stored at −80°C. Sampling continued for up to 8 days until the patient was discharged from ICU, and the extended Glasgow outcome scale (GOSE) was assessed at six months following admission.

### Measurement of melatonin

Serum melatonin concentrations were measured using a commercially available melatonin competitive binding enzyme linked immunosorbent assay (ELISA) kit (IBL Immunological Laboratories, Hamburg, Germany) as previously described (20). The kit used has a minimum detection limit of 1.5 pg/mL with an intra-assay coefficient of variance of 3.0–11.4% and an inter-assay coefficient of variance range of 6.4–19.3%.

### Statistical analysis

For the purposes of interpretation, day 1 is calculated as the day of admission to the ICU, beginning at 0000 h. Serum melatonin was found to be well approximated by a lognormal distribution so was log-transformed prior to analysis with results presented as geometric means [95% confidence intervals (CI)]. Longitudinal analysis of melatonin was performed using mixed linear modeling (PROC MIXED) with log-melatonin as the outcome and fixed main categorical effects for Group (A, B, or C), Time (0400 h, 1000 h, 1600 h, and 2200 h) and Day (1–8), with each patient treated as a random effect. To ascertain if time and day effects were consistent across groups, interaction between group and time and group and day were explored. Patient age and gender along with assay number were also examined but were not included in final models due to the lack of any apparent significant relationship with melatonin. All analysis was performed using SAS version 9.2 (SAS Institute, Inc., Cary, NC, USA).

## Results

### Patient characteristics

In total 45 patients were recruited into this study. Overall, the ages of the patients ranged from 16 to 86 years with a mean age of 42.5 ± 2.6 years. There was a male predominance, with a 34:11 ratio of males to females. Patients were enrolled in the study for up to 8 days from ICU admission and occurrence of TBI. As described, patients were recruited into three arms of the trial: patients admitted to the ICU with severe TBI defined as a GCS score of <9; patients admitted to the ICU having sustained trauma but without severe TBI; and patients admitted to the ICU without a history of trauma or severe TBI (see Table 1). Patients with trauma but not TBI sustained injuries including fractures of the long bones, vertebrae, or facial bones; pneumothoraces; pulmonary and cardiac contusions; intra-abdominal visceral injuries; and soft tissue injuries including lacerations and contusions. Patients in this group included those who had sustained intracranial injuries but had a GCS of at least 9. Patients admitted to ICU without trauma or TBI demonstrated conditions including infective endocarditis, perforated abdominal viscera, pneumonia, acute myocardial infection, and exacerbation of chronic conditions including asthma.

All patients in this study underwent individually patient-centered treatment in the ICU. There were no alterations in treatment afforded these patients due to their inclusion in this study. As part of their standard therapeutic regimens, they might have been administered any of barbiturate therapy, anti-epileptic therapy, non-steroidal anti-inflammatory medications, pharmacological sedation, and induced coma. Of note, the ICU does have different lighting conditions depending upon day/night, with a lower level of ambient lighting during the night.

### Severe TBI

Over the study period, 18 patients having sustained severe TBI were included in the TBI arm. There were 14 males and 4 females, with a mean age of 35.1 ± 3.2 (16–73). The mechanisms for injury included predominantly motor vehicle accidents (6, 33%), followed by motorcycle accidents (2, 11%), pedestrian (2, 11%), fall from height >2 m (1, 6%), assault (1, 6%), other (5, 28%), and one unknown (6%). There was a range of GCS with a mean of 5.5 ± 0.8.

**Figure 1:**
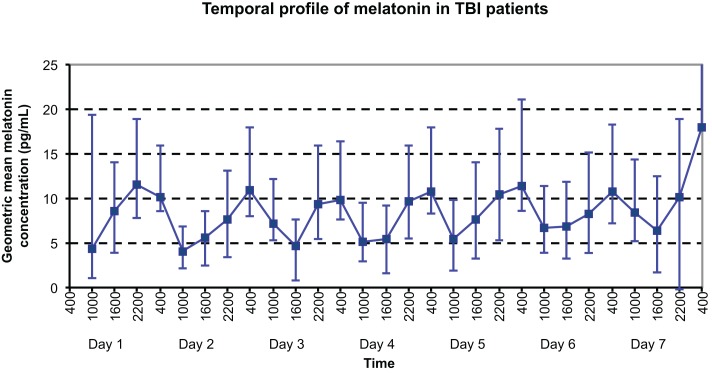
**Temporal profile of geometric mean serum melatonin concentrations in TBI patients, with peaks at 2200 h on Day 1 and 0400 h on Day 8, and nadir at 1000 h on Day 4**.

The temporal profile of serum melatonin for this group is shown in Figure 1, with geometric mean concentrations (95% CI) displayed for each timepoint measured. Daily maximum values were demonstrated as might be expected at night time: 2200 h on day 1 and 0400 h on all other days; whereas local minimum melatonin concentrations were evidenced during the daytime at 1000 h on all days apart from day 3, when a minimum was demonstrated at 1600 h.

### Trauma without severe TBI (trauma)

Fourteen patients who sustained traumatic injuries without severe TBI were included in the study. In this group, 14 suffered multitrauma, with those sustaining TBI demonstrating a GCS score ≥9. There were 13 males and 1 female, exhibiting a mean age of 50.7 ± 4.9 years (21–86 years). Again, motor vehicle accidents were most common in this group, occurring in 8 (57%) of patients, followed by motorcycle accidents (2, 14%), fall from height (1, 7%), assault (1, 7%), cyclist (1, 7%), and other (1, 7%). The mean GCS was 13.2 ± 0.7.

**Figure 2:**
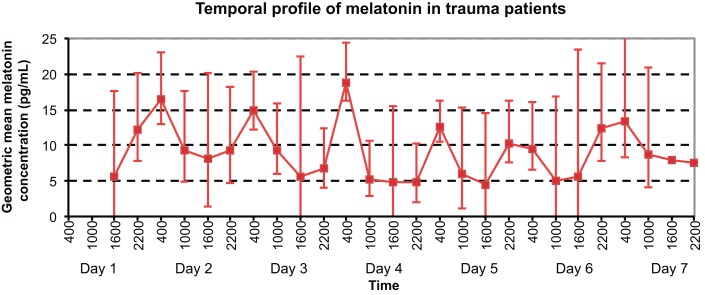
**Temporal profile of geometric mean serum melatonin concentrations in trauma patients without TBI, with peaks at 0400 h on Days 2, 3, 4, 5, and 7, and nadir at 1600 h on Day 5**.

As shown in Figure 2, daily maximum melatonin levels were demonstrated at 0400 h on days 2–5, 7, and at 2200 h on day 6. Local minimum values were exhibited at 1000 h on days 1–3, 6–7, at 1600 h on day 5, and at 2200 h on day 4.

### ICU without trauma or TBI

Over the study period, 13 patients treated in ICU without having sustained any form of head injury or trauma were included in the third group. There were seven males and six females, with a mean age of 43.9 ± 4.9 (22–74). Three of these patients sustained neurosurgical insults with two patients presenting with a World Federation of Neurological Surgeons grade V subarachnoid hemorrhage, and one with an intracerebral hemorrhage, with no history of trauma.

**Figure 3:**
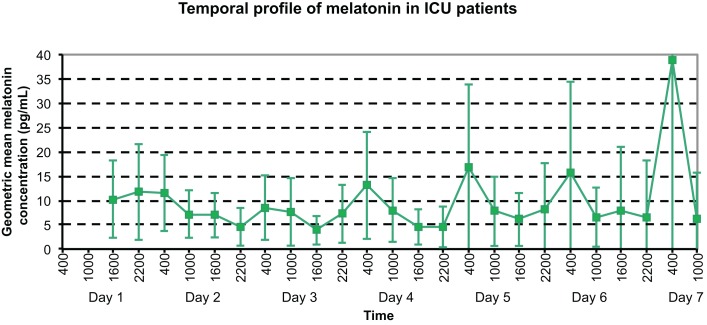
**Temporal profile of geometric mean serum melatonin concentrations in ICU patients, with peaks at varying times on the initial 3 days, then peaks at 0400 h on days 4–7**.

The temporal profile of serum melatonin for this group (see Figure 3) demonstrates geometric mean concentrations (95% CI) displayed for each timepoint measured. Daily maximum values were demonstrated at 2200 h on day 1, and 0400 h on all other days; whereas minimum melatonin concentrations were evidenced at 2200 h on days 2 and 6, 1600 h on days 3–5, and 1000 h on day 7.

### Control patients

**Figure 4:**
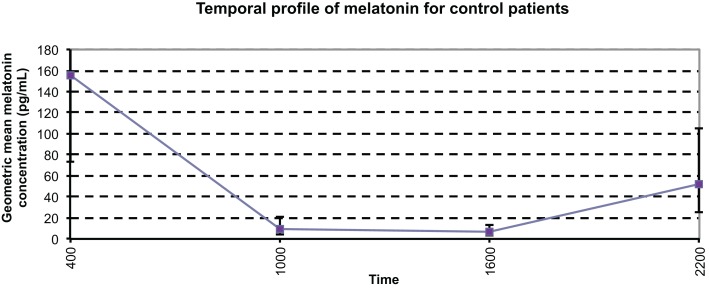
**Temporal profile of mean serum melatonin concentrations in controls, with a diurnal variation displaying maximum values at night, and minimum values during the day (*p* < 0.0001)**. The geometric mean concentrations for the control patients are significantly higher than those of the other patient groups.

In order to provide appropriate comparisons, six healthy people were recruited as control patients for this study. The control patients were healthy volunteers with no neurological conditions and on no medications, recruited from our laboratory. The control patients had serum samples taken every 6 h for a single 24-h period. The temporal profile of control patients is shown in Figure 4. The geometric mean serum melatonin concentrations ranged from 6.6 to 155.6 pg/mL with an overall geometric mean value of 27.0 (16.3–44.6) pg/mL. This range of serum melatonin concentrations differs somewhat to those reported in the literature of 7.4–82.5 (11, 21, 22) and the mean control serum melatonin concentration of 15.15 ± 1.65 pg/mL that this group has reported previously (20). However, it is important to note that Rizzo et al. (21) demonstrated melatonin concentrations ranging up to 163 pg/mL (21), and there is very little consistency in terms of sample collection methodology and analytical techniques among the studies we found in the literature. As expected under physiological conditions, endogenous melatonin concentrations were at a peak at 0400 h, with a mean value of 155.6 pg/mL, decreasing to daytime lows of 8.8 pg/mL at 1000 h and 6.6 pg/mL at 1600 h, before again rising at 2200 h. The profile of melatonin in this group coincides with normal physiological fluctuations in a day cycle.

### Comparisons of all ICU patient groups

Overall, when comparing the three groups, there was no statistically significant difference between the geometric mean melatonin concentrations analyzed via generalized linear modeling (*p* = 0.87). Regarding the CI of each group, there was little difference between the groups, with trauma and ICU both demonstrating a 95% CI of 5.3 pg/mL, and TBI patients a slightly smaller (yet not significant) 95% CI of 4.7 pg/mL, suggesting that a consistently homogenous variation of melatonin production across the groups. The geometric mean melatonin concentrations of each of the patient groups were demonstrated to be significantly lower than control patients (*p* < 0.0001).

**Figure 5:**
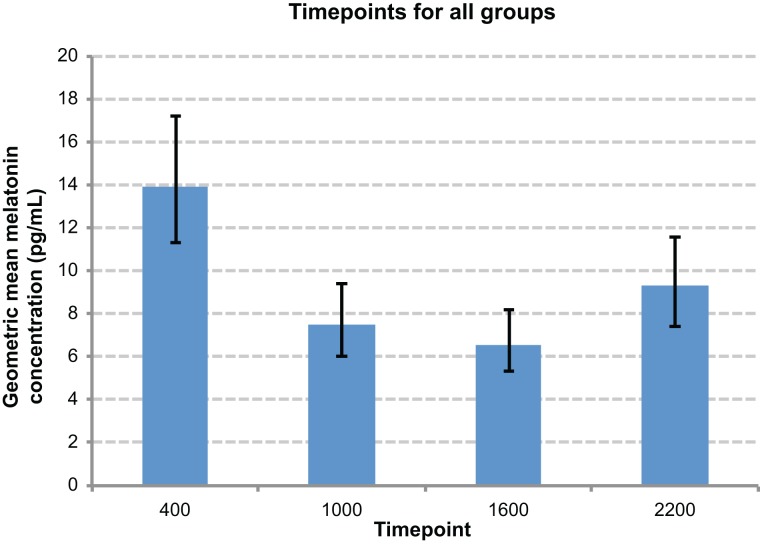
**Comparison graph of time point effect**. Night-time timepoints (0400 h and 2200 h) were significantly elevated compared to daytime time points, and 0400 h significantly higher than 2200 h.

**Figure 6:**
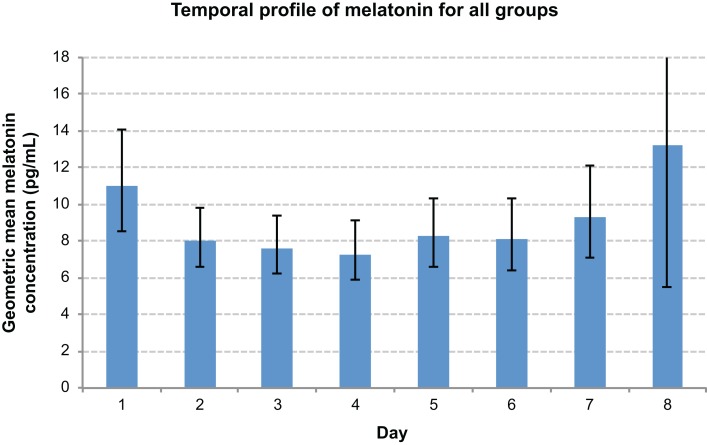
**Comparison graph of day effect**. Day 1 values were significantly elevated when compared with days 2–6.

There was an overall time effect (*p* < 0.0001), with the highest melatonin concentrations tending to occur at 0400 h, and the lowest at 1600 h (Figure 5). There was a day effect demonstrated, with day 1 most commonly exhibiting significantly higher melatonin concentrations than other days (*p* < 0.05) (Figure 6). An interaction between groups and timepoints was investigated, attempting to determine whether the time effect differed between groups, although this was not significant (*p* = 0.11). Similarly, there was no significant interaction between group and day (*p* = 0.56).

## Discussion

This study demonstrated a marked disruption of the diurnal rhythm in the TBI patients and the ICU patients, which resumed its normal pattern by the third day. Physiological studies have demonstrated that melatonin levels peak between 0200 h and 0400 h (11), and decrease toward their nadir during the day. However, in our study derangement of this rhythm was noted, in particular, during the first days measured.

Patients having sustained trauma but not TBI demonstrated a consistent peak melatonin concentration with a similar level at 0400 h on each day of the study, with the sole exception of day 6. The values of this group may thus be generalized as maintaining their circadian rhythm, with peaks occurring at 0400 h on each day. In contrast to trauma patients, both TBI and ICU patient groups demonstrated a clear disruption to the physiological pattern of melatonin concentrations. Patients with TBI first restored a maximal melatonin level on day 2 at 0400 h, albeit lower than the previous timepoint of 2200 h on day 1. This maximal level was also lower than the physiological levels at this time of 149 pg/mL in our controls and 163 pg/mL in the literature, after which the peak was maintained at 0400 h on each successive day of the study. Similarly, while the maximal daily melatonin concentration first occurred on day 2 in the ICU patient group, true restoration of the diurnal melatonin fluctuation did not take place until day 4.

It has already been established that the circadian rhythm of melatonin production is disrupted following both TBI and ICU admission (5, 8). However, what has not been elucidated, to date, to our knowledge, is whether trauma patients without severe TBI share this derangement or whether they remain relatively unaffected. From our data, it would appear that serum melatonin concentrations for patients who have sustained significant trauma (enough to warrant ICU admission) maintain their pattern of melatonin levels and thus a normal circadian rhythm.

Furthermore, our data suggest that a disruption of circadian rhythm of melatonin concentrations in the TBI patients. While demonstrating a maximal concentration at 0400 h on day 2, these patients only appear to “correct” their pattern by day 3. Many theories have been postulated as to the mechanisms behind this derangement, in view of melatonin’s effects as a neuroprotective agent. It may be postulated that imbalance in melatonin production and consumption, as well as any alteration of pineal gland function is a complex sequel of the general neuroendocrine dysfunction detected in TBI patients that affects the pituitary, hypothalamus, and their axes (23). Whether melatonin may be produced in response to the injury, depleted as it exerts its neuroprotective effects, or is further produced as a result of this depletion is unknown, and the design of this study does not allow for investigation of these hypotheses. However, it remains plausible that the consumption of endogenous melatonin in the attempt to counteract deleterious sequelae of TBI may disrupt the physiological circadian rhythm which, in turn, may result in the sleep disturbances witnessed in TBI patients (5).

Similarly, ICU patients exhibit alteration of expected melatonin profiles until day 3. Potential factors involved in this disruption include performance of medical procedures at all hours, and high noise levels, as well as the continuous monitoring of vital signs (8). In many ICUs, high intensity of ambient light is present even at night, however, in the ICU in which the study was undertaken, there is decreased light during nocturnal hours to respect a more physiological day/night rhythm. Despite this, there remain factors, which might influence the physiological production of melatonin in this setting. As such, this derangement would be anticipated to continue at least until the patients had been discharged from the ICU, however, this was witnessed only until day 3 after which time the rhythm was restored but not the physiological concentrations of melatonin. It may be postulated that following the initial disruption to the circadian rhythm, the body resynchronizes its melatonin production accordingly, though the mechanisms leading to this phenomenon remain obscure.

There were some interesting characteristics borne out in the temporal profiles. All of the three groups demonstrated similar minimum concentrations, with the minimum geometric mean melatonin concentrations varying by 0.7 pg/mL between the three groups. However, the maximum levels demonstrated greater variation of 20.9 pg/mL across the groups, with TBI patients and trauma patients having similar maximum geometric mean melatonin concentrations, and ICU patients a greater maximum value. When looking specifically at the 95% CI, all groups were similar with TBI patients demonstrating a range of 4.7 pg/mL, and both trauma and ICU patients 5.3 pg/mL.

The values exhibited in TBI and trauma patients when contrasted against ICU patients, support the potential impact of trauma in endogenous melatonin concentrations. While not clearly understood, it appears that melatonin levels are altered in these settings. These derangements have been previously attributed to conditions such as the ambient lighting in ICU and the continual disruption of patients’ sleep by medical, nursing, and monitoring needs. This study has not been designed to investigate the precise causative factors for these derangements.

Our study demonstrates that all groups exhibit lower melatonin concentrations than control patients. This finding is in contrast with our previous study, which demonstrated that single daily measurements of serum melatonin levels were not significantly increased in TBI comparative to controls (20), though it may be argued that this study is more appropriately designed to identify these differences. The precise pathophysiological processes underlying the decreases in serum melatonin for all our patient groups is unclear, and it may, in fact, be that all patients with significant illness have decreased melatonin, be it as a result of increased consumption, lack of production, or a combination of the two. However, it must be noted that there is great difficulty in performing accurate comparisons between our current and previous studies. There was a significant difference in our sampling techniques and times between the two studies (the current study comprise four separate sampling timepoints of serum, whereas the previous study comprised a single sampling at 0900 h) (20).

Sleep disturbances (4) and, in fact, delayed circadian rhythms have been reported in patients with mild TBI (24) and also severe TBI (5). In a study performed by Shekleton et al., salivary concentrations of endogenous melatonin were collected overnight every half hour in patients with TBI, and correlated with polysomnographic sleep recording. Patients with TBI demonstrated sleep disturbances as well as decreased melatonin concentrations overnight (5). Ponsford et al. showed long-term deficiency of sleep patterns associated with fatigue in TBI patients (6). To this end, a clinical trial is currently underway wherein children suffering from post-concussion syndrome receive sublingual melatonin for a month post-event (25) in an attempt to reduce symptoms following TBI. It might be that the degree of TBI impacts particularly on the circadian rhythm, though this remains speculation at this point in time.

While this study aimed to provide a temporal profile of endogenous melatonin concentrations throughout the duration of a patient’s admission to ICU, the maximum length of stay was only 8 days. The collection of serum samples at four time points of each day was able to afford detailed insight into the variation of levels throughout a 24-h period; however, it may be inadequate in duration for investigating the response of endogenous melatonin over a longer time period. A further confounding factor might be the inclusion of patients with brain injury in the ICU patient group. Despite these patients not having sustained TBI, they nonetheless did suffer brain injury, and this may have impacted upon the melatonin concentrations in these patients. Furthermore, owing to the small group sizes, there is a degree of increased variation in values. Similarly, our control patient values demonstrated significant variation between day and night time values, which might have been addressed by increased control patient numbers. Despite these limitations to our study, we feel that this work is able to contribute to an understanding of the temporal profile of melatonin following TBI and in the ICU patient.

## Conclusion

This study aims to further elucidate the temporal profile of melatonin in patients with severe TBI, trauma without severe head injury, and ICU patients. There are derangements of the circadian rhythm of melatonin production in patients undergoing severe head injury, and decreased concentrations in patients with both severe TBI and trauma without severe head injury when compared to controls. It remains likely that these derangements may be due, in part, to alterations in melatonin production and/or metabolism, or to the deleterious effects of oxidative stress and the physiological damage imposed by TBI. This study provides valuable data regarding temporal profiles of melatonin, which might, in the future, be used to target therapeutic potentials of melatonin administration in patients having sustained severe TBI.

## Conflict of Interest Statement

The authors declare that the research was conducted in the absence of any commercial or financial relationships that could be construed as a potential conflict of interest.

## References

[1] Hukkelhoven CW Steyerberg EW Rampen AJ Farace E Habbema JD Marshall LF Patient age and outcome following severe traumatic brain injury: an analysis of 5600 patients. J Neurosurg (2003) 99:666–73. 10.3171/jns.2003.99.4.0666 14567601

[2] Roozenbeek B Maas AI Menon DK. Changing patterns in the epidemiology of traumatic brain injury. Nat Rev Neurol (2013) 9:231–6. 10.1038/nrneurol.2013.22 23443846

[3] Ouellet M Beaulieu-Bonneau S Morin C. Insomnia in patients with traumatic brain injury: frequency, characteristics, and risk factors. J Head Trauma Rehabil (2006) 21:199–212. 10.1097/00001199-200605000-00001 16717498

[4] Cohen M Oksenberg D Snir D Stern M Groswasser Z. Temporally related changes of sleep complaints in traumatic brain injured patients. J Neurol Neurosurg Psychiatry (1992) 55:313–5. 10.1136/jnnp.55.4.313 1583518 PMC489047

[5] Shekleton J Parcell D Redman J Phipps-Nelson J Ponsford J Rajaratnam S. Sleep disturbance and melatonin levels following traumatic brain injury. Neurology (2010) 74:1732–8. 10.1212/WNL.0b013e3181e0438b 20498441 PMC3462582

[6] Ponsford J Ziino C Parcell D Shekleton J Roper M Redman J Fatigue and sleep disturbance following traumatic brain injury – their nature, causes and potential treatments. J Head Trauma Rehabil (2012) 27:224–33. 10.1097/HTR.0b013e31824ee1a8 22573041

[7] Shilo L Dagan Y Smorjik Y Weinberg U Dolev S Komptel B Effect of melatonin on sleep quality of COPD intensive care patients: a pilot study. Chronobiol Int (2000) 17:71–6. 10.1081/CBI-100101033 10672435

[8] Shilo L Dagan Y Smorjik Y Weinberg U Dolev S Komptel B Patients in the intensive care unit suffer from severe lack of sleep associated with loss of normal melatonin secretion pattern. Am J Med Sci (1999) 317:278. 10.1097/00000441-199905000-00002 10334113

[9] Reiter RJ. Melatonin: clinical relevance. Best Pract Res Clin Endocrinol Metab (2003) 17:273–85. 10.1016/S1521-690X(03)00016-2 12787552

[10] Rajaratnam S Cohen D Rogers N Melatonin and melatonin analogues. Sleep Med Clin (2009) 4:179–93 10.1016/j.jsmc.2009.02.007

[11] Brzezinski A Mechanisms of disease: melatonin in humans. N Engl J Med (1997) 336:186–95 10.1056/NEJM199701163360306 8988899

[12] Lewy A Emens J Jackman A Yuhas K. Circadian uses of melatonin in humans. Chronobiol Int (2006) 23:403–12. 10.1080/07420520500545862 16687313

[13] Longoni B Salgo MG Pryor WA Marchiafava PL. Effects of melatonin on lipid peroxidation induced by oxygen radicals. Life Sci (1998) 62:853–9. 10.1016/S0024-3205(98)00002-2 9496707

[14] Rodriguez C Mayo JC Sainz RM Antolin I Herrera F Martin V Regulation of antioxidant enzymes: a significant role for melatonin. J Pineal Res (2004) 36:1–9. 10.1046/j.1600-079X.2003.00092.x 14675124

[15] Samantaray S Das A Thakore NP Matzelle DD Reiter RJ Ray SK Therapeutic potential of melatonin in traumatic central nervous system injury. J Pineal Res (2009) 47:134–42. 10.1111/j.1600-079X.2009.00703.x 19627458 PMC11877319

[16] Sarrafzadeh AS Thomale U-W Kroppenstedt S-N Unterberg AW Neuroprotective effect of melatonin on cortical impact injury in the rat. Acta Neurochir (2000) 142:1293–9 10.1007/s007010070028 11201646

[17] Gorgulu A Palaoglu S Ismailoglu O Tuncel M Surucu MT Erbil M Effect of melatonin on cerebral edema in rats. Neurosurgery (2001) 49:1434–42. 10.1097/00006123-200112000-00024 11846944

[18] Beni SM Kohen R Reiter RJ Tan D-X Shohami E. Melatonin-induced neuroprotection after closed head injury is associated with increased brain antioxidants and attenuated late-phase activation of NF-kB and AP-1. FASEB J (2004) 18:149–51. 10.1096/fj.03-0346fje 14597558

[19] Ates O Cayli S Gurses I Yucel N Iraz M Altinoz E Effect of pinealectomy and melatonin replacement on morphological and biochemical recovery after traumatic brain injury. Int J Dev Neurosci (2006) 24:357–63. 10.1016/j.ijdevneu.2006.08.003 16959465

[20] Seifman M Adamides A Nguyen P Vallance S Cooper D Kossmann T Endogenous melatonin increases in cerebrospinal fluid of patients after severe traumatic brain injury and correlates with oxidative stress and metabolic disarray. J Cereb Blood Flow Metab (2008) 28:684–96. 10.1038/sj.jcbfm.9600603 18183032

[21] Rizzo V Porta C Moroni M Scoglio E Moratti R. Determination of free and total (free plus protein-bound) melatonin in plasma and cerebrospinal fluid by high-performance liquid chromatography with fluorescence detection. J Chromatogr B Analyt Technol Biomed Life Sci (2002) 774:17–24. 10.1016/S1570-0232(02)00168-X 12052718

[22] Paparrigopoulos T Melissaki A Tsekou H Efthymiou A Kribeni G Baziotis N Melatonin secretion after head injury: a pilot study. Brain Inj (2006) 20:873–8. 10.1080/02699050600832114 17060154

[23] Agha A Rogers B Mylotte D Taleb F Tormey W Phillips J Neuroendocrine dysfunction in the acute phase of traumatic brain injury. Clin Endocrinol (2004) 60:584–91. 10.1111/j.1365-2265.2004.02023.x 15104561

[24] Ayalon L Borodkin K Dishon L Kanety H Dagan Y. Circadian rhythm sleep disorders following mild traumatic brain injury. Neurology (2007) 68:1136–40. 10.1212/01.wnl.0000258672.52836.30 17404196

[25] Barlow K Brooks B Macmaster F Kirton A Seeger T Esser M A double-blind, placebo-controlled intervention trial of 3 and 10mg sublingual melatonin for post-concussion syndrome in youths (PLAYGAME): study protocol for a randomized controlled trial. Trials (2014) 15:271 10.1186/1745-6215-15-271 25001947 PMC4227124

